# Characterization and Formulation of Isoniazid for High-Dose Dry Powder Inhalation

**DOI:** 10.3390/pharmaceutics11050233

**Published:** 2019-05-13

**Authors:** Imco Sibum, Paul Hagedoorn, Henderik W. Frijlink, Floris Grasmeijer

**Affiliations:** Department of Pharmaceutical Technology and Biopharmacy, Faculty of Science and Engineering, University of Groningen, 9700 AB Groningen, The Netherlands; p.hagedoorn@rug.nl (P.H.); h.w.frijlink@rug.nl (H.W.F.); f.grasmeijer@rug.nl (F.G.)

**Keywords:** high dose pulmonary delivery, dry powder inhaler, drug formulation, inhalation, tuberculosis

## Abstract

Tuberculosis is a major health problem and remains one of the main causes of mortality. In recent years, there has been an increased interest in the pulmonary delivery of antibiotics to treat tuberculosis. Isoniazid is one of these antibiotics. In this study, we aimed to characterize isoniazid and formulate it into a dry powder for pulmonary administration with little or no excipient, and for use in the disposable Twincer^®^ inhaler. Isoniazid was jet milled and spray dried with and without the excipient l-leucine. Physiochemical characterization showed that isoniazid has a low Tg of −3.99 ± 0.18 °C and starts to sublimate around 80 °C. Milling isoniazid with and without excipients did not result in a suitable formulation, as it resulted in a low and highly variable fine particle fraction. Spray drying pure isoniazid resulted in particles too large for pulmonary administration. The addition of 5% l-leucine resulted in a fraction <5 µm = 89.61% ± 1.77% from spray drying, which dispersed well from the Twincer^®^. However, storage stability was poor at higher relative humidity, which likely results from dissolution-crystallization. Therefore, follow up research is needed to further optimize this spray dried formulation.

## 1. Introduction

Tuberculosis (TB) is a major worldwide health problem and remains one of the main causes of mortality [1]. The disease is difficult to treat, as mycobacterium tuberculosis infects poorly perfused areas of the body, and is able to survive in tubercles, granulomas, and alveolar macrophages. As a result, high doses of antibiotics are required to attain bactericidal concentrations at the infected sites. Furthermore, over 80% of TB infections remain localized in the lungs, and only a small fraction of orally or intravenously administered antibiotics reaches this infected site [2]. This leads to sub-bactericidal antibiotic concentrations at the sites of infection, which increase the chance of development of bacterial resistance, whereas relatively high systemic concentrations may lead to adverse drug reactions. Other, more targeted administration strategies could, therefore, have significant advantages in the treatment of TB [3].

One such targeted route of administration is the pulmonary route. Use of this route permits a higher fraction of the administered antibiotics to reach the infected lungs. As a result, higher local concentrations are attained, which may even exterminate bacteria that are considered resistant [4]. With the lower systemic exposure resulting from a more targeted approach, systemic side effects (such as oto- or hepatotoxicity) are reduced [2,3,4,5,6,7].

Currently, pulmonary administration of antibiotics is mostly performed by wet nebulization. However, this form of pulmonary administration is time-consuming, carries the risk of patient reinfection and bacterial resistance building in the device, and is generally less patient friendly than a dry powder inhaler (DPI) [3,8,9]. Furthermore, wet nebulization requires clean water to reconstitute powder or a cold-chain for transportation and storage of antibiotic solutions. Further nebulizers require electricity. So this method is not suited for use in many less-developed countries, where most cases of TB occur [1,4]. In contrast, a dry powder formulation in combination with a DPI would not require all these prerequisites to function and, therefore, is more suitable for large-scale usage in these areas.

No antibiotic DPI formulations for use in TB treatment are currently on the market. However, tobramycin and colistin DPI products are marketed for use in the treatment of cystic fibrosis. The tobramycin-containing TOBI^®^ Podhaler^®^ uses PulmoSphere™ technology designed to enable administration of drugs independent of their physicochemical properties. While this technology results in efficient dispersion, the high excipient fraction and multi-step processing make this formulation type expensive and less suitable when high doses of antibiotics (such as those used in the treatment of TB) are needed. Furthermore, the Podhaler^®^ is a multi-use capsule inhaler, which, like all multi-use inhalers, introduces the risk of patient reinfection [10]. The Colobreathe^®^, a colistin containing product, also uses a multi-use capsule inhaler [11]. A more appropriate method for treatment of TB might be an antibiotic formulation with little or no excipient and simple processing in a cheap, disposable inhaler [3].

A prime candidate for inhalation therapy in TB is isoniazid. Isoniazid is one of the most potent anti-tuberculosis drugs [12]. Isoniazid is used in first line treatment and to prevent latent TB from progressing. However, resistance has been found worldwide in approximately 15% of the TB cases [13]. While the resistance mechanisms against isoniazid are multifold, the recommendation to overcome this resistance is straightforward: increase the dose of isoniazid [14]. As described above, the best approach to increase the concentration of the drug in the lungs is pulmonary administration.

To date, the challenge of formulating isoniazid in an appropriate dry powder for pulmonary administration has received scant attention in the research literature. Sawatdee formulated isoniazid using spray drying and physical mixing with a sugar carrier in a ratio of 1:1.67 to get a powder suitable for inhalation therapy [15]. Rojanarat produced isoniazid–proliposome powders for pulmonary administration. However, the drug load was quite low (<10%) and the formulation resulted in a relatively low maximum fine particle fraction (FPF) of 35% [16]. As isoniazid has to be given in the hundreds of milligrams range, the suitability of these two formulations is questionable. More research is required to formulate isoniazid in an appropriate dry powder.

In this study, we aimed to formulate isoniazid in a dry powder for pulmonary administration with little or no excipient for use in the cheap and disposable Twincer^®^ inhaler. A first step towards this goal is the extensive physicochemical characterization of isoniazid. Subsequently we evaluated the use of air jet milling and spray drying techniques, in combination with dispersion and stabilization aids, as a simple means to obtain a suitable dry powder inhalation formulation.

## 2. Materials and Methods

### 2.1. Materials

Isoniazid, l-leucine, and magnesium stearate were obtained from Sigma-Aldrich (Steinheim, Germany). Isoniazid was ordered in two batches, batch #075K1581 and #MKBV9475V, referred to in this article as B1 and B2, respectively.

### 2.2. Light Microscopy

Starting materials were imaged using a Nikon SMZ-U light microscope, obtained from Nikon Instruments (Tokyo, Japan). Crystals were placed on the sample stage without being fixed. A SC100 camera, obtained from Olympus (Tokyo, Japan), was used to take the images.

### 2.3. Scanning Electron Microscopy

Samples were imaged using a JSM 6460 scanning electron microscope (SEM), supplied by JEOL (Tokyo, Japan). Samples were fixed on sample stubs by the use of double-sided adhesive carbon tape and sputter coated with 10 nm of gold using a JFC-1300 auto fine coater purged with argon gas. Imaging took place under high vacuum, with a spotsize of 25, an acceleration voltage of 10 kV, and a working distance of 10 mm. The secondary electron detector was used for imaging.

### 2.4. Differential Scanning Calorimetry

Differential scanning calorimetry (DSC) was performed with the Q2000 supplied by TA instruments (New Castle, PA, USA). A sample of 2 to 5 mg was placed in a Tzero pan and hermetically sealed. To ascertain the crystallinity of the powders, pans were loaded into the DSC at 20 °C and a temperature ramp from 20 °C until 210 °C was made at a rate of 20 °C/min. Samples were always measured the same day as they were spray dried or milled and each sample was measured in duplicate. Tg was determined with quench cooling. The same heating ramps were used, the cooling ramp ran from 210 °C to −80 °C at a rate of 100 °C/min. Data were analyzed in Universal analysis 4.5.0.5. Tg was defined as the inflection point in the step transition on the second heating.

### 2.5. Thermogravimetric Analysis

Thermogravimetric analysis (TGA) was performed on the isoniazid starting material with the Discovery TGA supplied by TA instruments (New Castle, PA, USA). Six to eight milligrams of sample were placed on a platinum pan. The pan was heated from 80 °C to 140 °C in steps of 20 °C. After each step, the temperature was kept stable for four hours. Weight loss percentage over these four-hour periods was then calculated.

### 2.6. Milling

Milling was performed using a 50 AS spiral jet mill equipped with a 0.8 mm nozzle obtained from Hosokawa Alpine (Augsburg, Germany). Three different milling pressures were tested: 7 bar nozzle and 2 bar mill; 5 bar nozzle and 2 bar mill; and lastly, 4 bar nozzle and 0.5 bar mill. Nitrogen was used as gas. Isoniazid was milled in its pure form, with the addition of magnesium stearate in concentrations of 0.1%, 0.5%, 1%, and 2% *w*/*w*, or with l-leucine in concentrations of 0.5%, 1%, 2%, and 5% *w*/*w*. The powders that contain magnesium stearate or l-leucine were only milled at 4 bar nozzle and 0.5 bar mill pressures.

### 2.7. Spray Drying

Spray drying was performed using a Büchi B-290 mini spray dryer in closed loop configuration, equipped with a high-performance cyclone, a B-296 dehumidifier, and a B-295 inert loop (Flawil, Switzerland). A range of parameters was investigated, presented in Table 1. All samples were produced in duplicate. Condition 1 was also used to spray dry a formulation with 5% l-leucine *w*/*w*, referred to as sample LEU. Isoniazid was always dissolved in demineralized water right before spray drying. Before spray drying the solutions, the outlet temperature was stabilized by spraying demineralized water into the system.

### 2.8. Laser Diffraction Analysis

Primary particle sizes were measured using a HELOS BF diffraction unit with a RODOS dry disperser, both obtained from Sympatec (Clausthal-Zellerfeld, Germany). The HELOS diffraction unit was equipped with an R3 lens, allowing a measurement range of 0.1 μm to 175 μm. Powders were dispersed with the RODOS at 0.5, 3, and 5 bars. All measurements were performed twice for the milled samples and three times for the spray dried samples. The average with standard deviation was calculated from the results.

### 2.9. Inhaler Dispersion

Dispersion measurements were performed using the HELOS diffraction unit equipped with the INHALER 2000 adaptor, also obtained from Sympatec (Clausthal-Zellerfeld, Germany). The Twincer^®^ DPI was used for dispersion. Measurements were performed at pressure drops of 2, 4, and 6 kPa. The dose tested was 25 mg, as more milled isoniazid did not fit into the dose compartment of the inhaler. Fine particle fraction (FPF) was calculated as a percentage of the delivered dose. All samples were measured twice.

### 2.10. Storage Stability

To determine the effect of storage on the primary particle size distribution, ten separate 5% l-leucine *w*/*w* containing batches were spray dried. These batches were stored in pairs in desiccators at relative humidities (RHs) of 0%, 32%, 58%, 75%, and 90% and a temperature of 25 °C for 48 h.

### 2.11. X-Ray Diffraction Analysis

A Bruker D2 Phaser (Billerica, United States) was used to perform X-ray analysis. The scanning range was 5 to 60°2θ width of 0.004°2θ and a step time of 1 s. The detector opening was 5°2θ. The divergence slit and air scatter screen were 1 mm and 3 mm, respectively. The sample stage spun at 60 rpm during measurements.

### 2.12. Dynamic Vapor Sorption

For dynamic vapor sorption (DVS) analysis, three separate samples were spray dried. The inlet temperature during spray drying was changed to 120 °C and the feed rate to 1 mL/min. The atomizing and aspirator airflow were kept the same. Samples contained either 0%, 2%, or 4% l-leucine. DVS analysis was performed on the same day of spray drying. The change in spray dry settings was made to try and improve the efficiency of the l-leucine coating around the isoniazid core. The percentages of 2% and 4% were chosen to see the effect of different percentages of l-leucine on the absorption and desorption spectra.

A DVS-1000, supplied by Surface Measurement Systems (Middlesex, United Kingdom), was used to perform sorption and desorption measurements. Approximately 45 mg of sample was used for analysis. The run was started at 0% RH and only increased to the next step after the mass change per time interval (dm/dt) was lower than 0.0005%/min, indicating that the mass had stabilized. The RH was then increased in steps of 30% to a maximum of 90% RH, after which RH was decreased in similar steps to 0% RH. The cycle was then repeated.

### 2.13. Inline Laser Diffraction Analysis during Spray Drying

To establish the particle size distribution of the powder before collection, the B-290 spray dryer was modified to allow for inline laser diffraction analysis during the spray drying process. Air of the drying chamber was passed with tubing to the INHALER2000 adapter attached to the HELOS BF diffraction unit (Sympatec, Clausthal-Zellerfeld, Germany). The HELOS diffraction unit was equipped with an R3 lens, allowing a measurement range of 0.1 μm to 175 μm. Five measurements of 10 s each were made at 20 s intervals. The outlet of the INHALER2000 adapter was attached with tubing to the cyclone of the B-290 spray dryer, which collected the powder. Normal laser diffraction analysis (see section ‘Laser diffraction analysis’) was performed on the collected powder. Condition number 1 was adopted as the spray dry setting.

## 3. Results

### 3.1. Physicochemical Characterization

**Figure 1:**
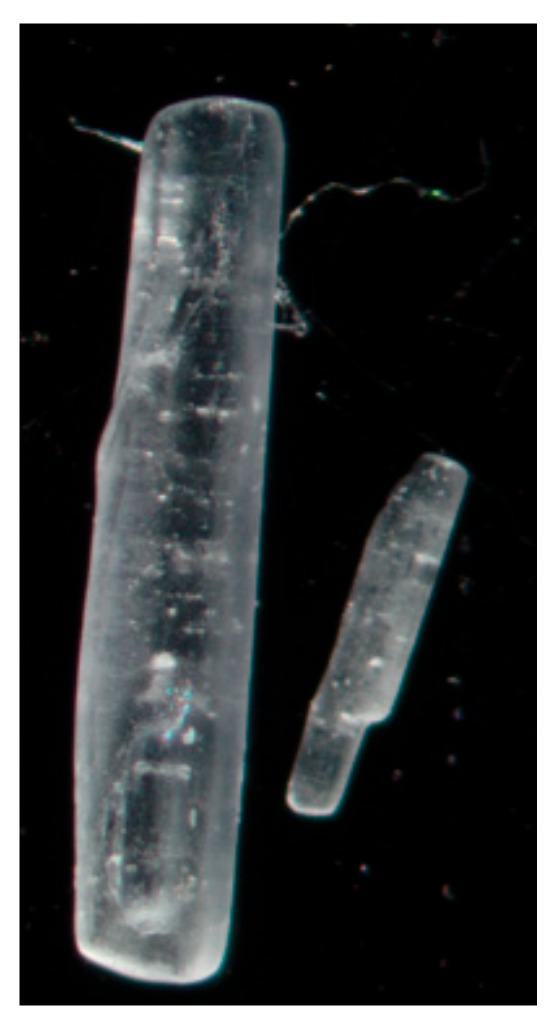
Light microscope image of two representative isoniazid crystals, B1 left and B2 right. Magnification: 2.5×.

Figure 1 shows representative crystals from the two different batches used; the B1 batch on the left and the B2 batch on the right. As can be seen, the B1 batch has substantially bigger crystals than the B2 batch.

**Figure 2:**
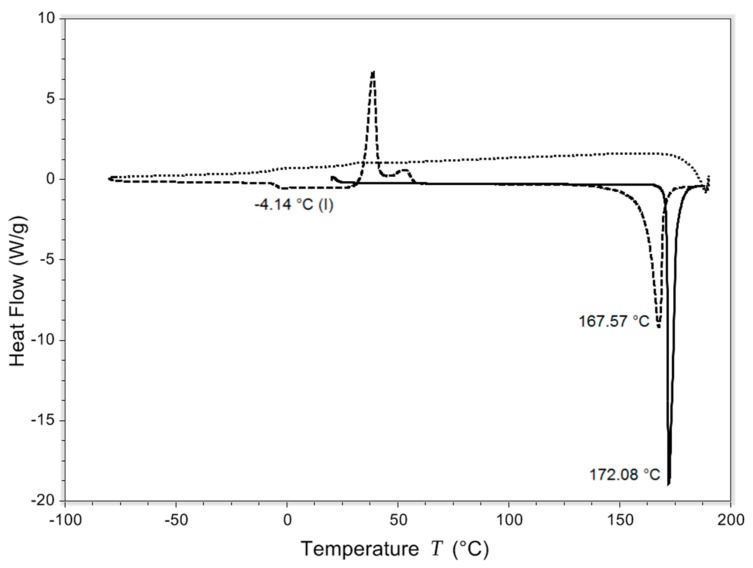
Differential scanning calorimetry (DSC) curve of one of the heat/quench cool/heat experiments. The solid line is the first heating, the short dashed line is quench cooling, and the long dashed line second heating. Exo is up.

Isoniazid has a Tg of −4.14 °C in the DSC run shown in Figure 2. In total, four such runs were realized with an average Tg of −3.99 ± 0.18 °C. The melting temperature was found to be 172.23 ± 0.20 °C. After spray drying or milling, all samples were crystalline (Figure S1).

TGA was used to show mass loss in four-hour periods at a specific temperature. At 80 °C, barely any mass loss was seen with a decrease of only 0.06% ± 0.02%. As Table 2 shows, the rate of mass loss increased with increasing temperature.

### 3.2. Milling

**Figure 3:**
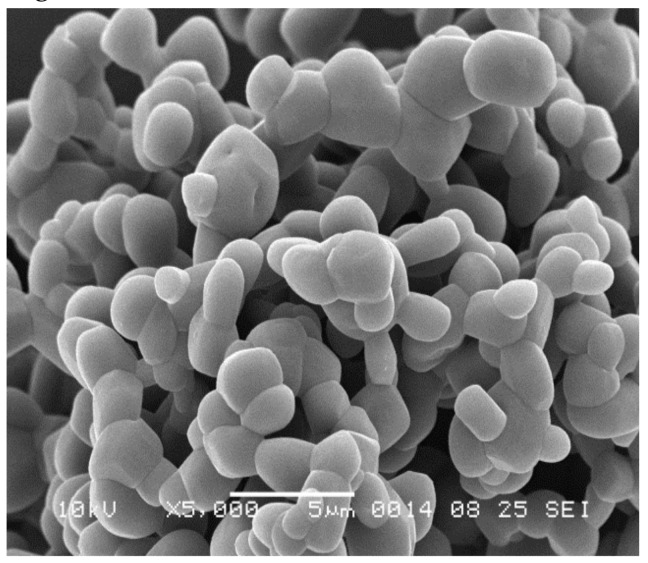
Representative scanning electron microscope (SEM) image of milled isoniazid without excipients. B2 batch. Magnification: 5000×.

The milled isoniazid had a fraction <5 μm varying from 94.53% to 100% for batch B1 with higher fractions at increasing milling pressures, while for the B2 batch this varied from 88.84% to 98.74%. SEM imaging (Figure 3) showed that milling isoniazid results in merely spherical particles of which many particles seem fused together.

**Figure 4:**
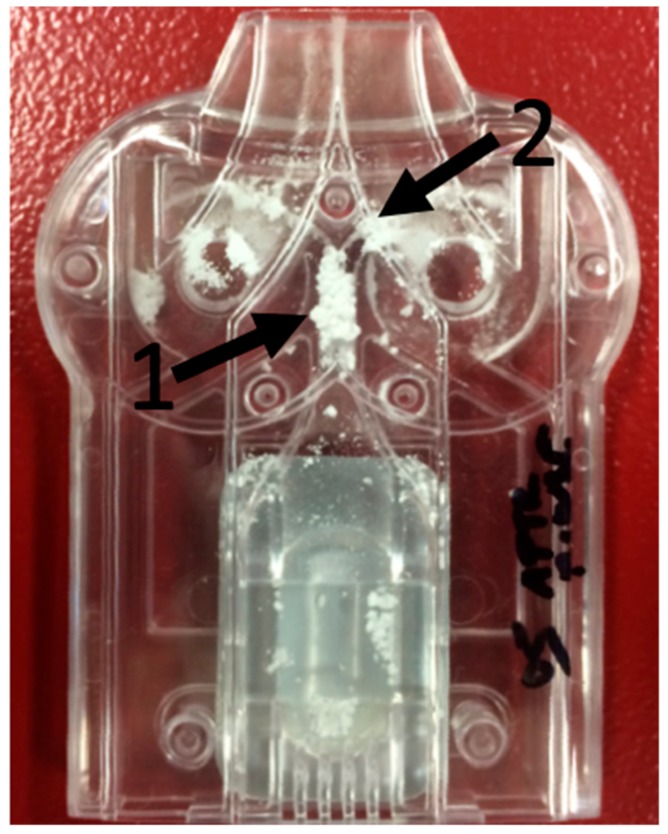
Representative picture of a blocked Twincer^®^ inhaler. Note the arrows pointing at the two areas where blockages occurred.

In the dispersion measurements, the B2 batch blocked the Twincer^®^ in more than half of the executed experiments, and no useful dispersion data could be obtained (Figure 4). The blockades occurred at two sites of the inhaler; the powder channel marked 1 and the classifier inlet channel marked 2. Blockades in the powder channel could be prevented by increasing the width of the channel. This, however, moved the problem to the classifier inlet channel. Increasing this channel width did not result in a drop in the occurrence of blockades. Batch B1 samples showed a maximum FPF of 28.36%, while the lowest retention found was still 25.07%.

**Figure 5:**
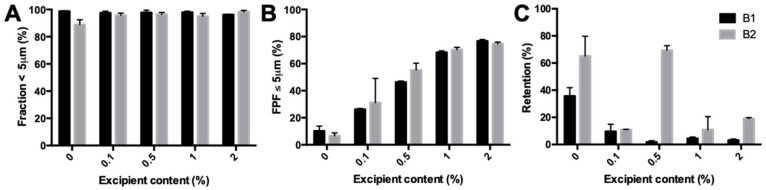
Primary particle size distribution and dispersion data of milled isoniazid with different fractions of magnesium stearate. (**A**) shows the fraction <5 μm found with the RODOS. (**B**) shows the fine particle fraction (FPF) found in the dispersion measurement with a dose of 25 mg and a pressure of 4 KPa. (**C**) is the retention found during those measurements.

The fraction <5 μm of isoniazid milled with different fractions of magnesium stearate varied between 94.95% and 98.17% (Figure 5A). Figure 5B shows the FPF found during the dispersion measurements. FPF increased almost threefold between 0.1% and 2% magnesium stearate. No major difference was found in the retention, except for B2 with 0.5% magnesium stearate, which did have an improved FPF, but no improvement in retention compared with pure isoniazid. All other batches had a retention significantly lower than that found for pure isoniazid.

**Figure 6:**
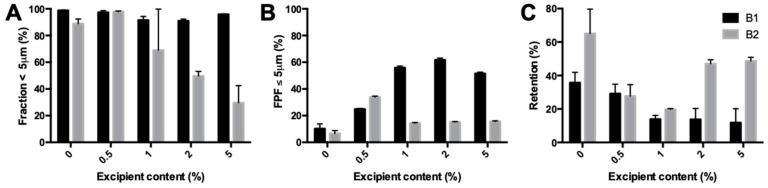
Primary particle size distribution and dispersion data of milled isoniazid with different amounts of l-leucine. (**A**) shows the fraction <5 μm found with the RODOS. (**B**) shows the FPF found in the dispersion measurement with a dose of 25 mg and a pressure of 4 KPa. (**C**) is the retention found during dispersion.

The fraction <5 μm of isoniazid milled with l-leucine showed a different trend for the two batches tested (Figure 6A). For batch B1, the fraction was similar for all samples. However, for batch B2, the fraction decreased significantly with increasing excipient content, from 97.89% ± 0.48% for 0.5% l-leucine down to 29.50% ± 12.94% for 5%. The FPF found during the dispersion measurement is shown in Figure 6B. The FPF increased between 0.5% and 2% leucine content for the B1 batch and decreased again at 5%. The retention (Figure 6C) decreased from 0.5% to 1% l-leucine and was constant after that. The B2 batch showed a decrease in FPF from 0.5% to 1% and after that was stable. Furthermore, the FPF from 1% to 5% l-leucine is much lower than for B1. At excipient contents higher than 0.5%, the B2 batch resulted in higher retentions compared with the B1 batch.

### 3.3. Spray Drying

**Figure 7:**
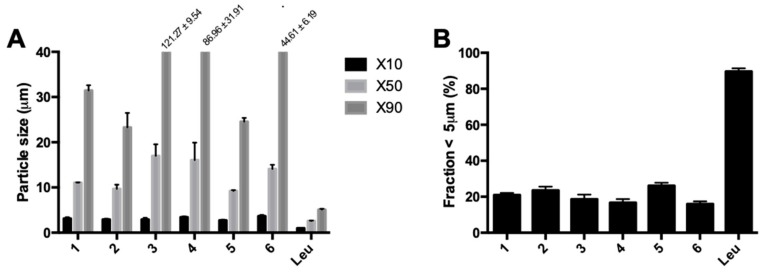
The laser diffraction data on the different samples spray dried in the B-290. Each sample was spray dried twice and each laser diffraction measurement was performed three times. (**A**) shows the X10, X50, and X90, while (**B**) shows the fraction <5 µm. The *x*-axis labels refer to the conditions applied (Table 1). Sample Leu is condition 1 with the addition of 5% l-leucine *w*/*w*.

Spray-dried isoniazid demonstrated uncharacteristically large particles for spray-dried material, as can be seen in Figure 7A,B. Contrary to expectations based on theory, changes in spray dry parameters did not change the result. The increase in feed rate between conditions 3 and 4 decreased the particle size distribution, while an increase was expected. The inlet temperature had the largest influence on particle size, with particle size increasing in parallel with temperature. The addition of 5% l-leucine, called Leu in Figure 7, led to a remarkable difference in the particle size distribution; that is, particles were mainly in an inhalable range (fraction < 5 µm = 89.61% ± 1.77%). The FPF found was 76.67% ± 0.13% with a retention of 9.45% ± 4.57%. Furthermore, 50 mg of spray-dried isoniazid could be filled in the inhaler, compared with only 25 mg of the milled material.

To ascertain if the large particles are formed during spray drying or after collection, the particle size distribution was measured between the drying chamber and the cyclone, before collection. The laser diffraction data of spray-dried pure isoniazid is shown in Table 3. The particles substantially increase in size after collection. Furthermore, the particle size distribution seen during spray drying is more comparable to what is expected based on theory and the droplets generated by the nozzle, which are around 27 μm in size [17].

**Figure 8:**
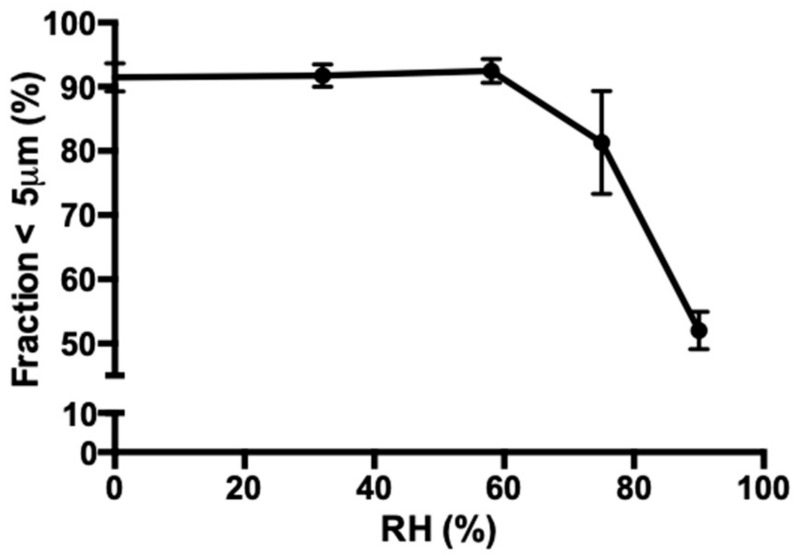
The change in the fraction <5 µm of the 5% l-leucine formulation as a result of storage at different relative humidities (RHs) at 25 °C for 48 h. Each sample was spray dried twice and each laser diffraction measurement was performed three times.

The fraction <5 µm of spray dried isoniazid with 5% l-leucine decreases significantly when stored at elevated relative humidity (Figure 8). The size of the particles was stable from 0% to 58% RH. However, at 75% and 90% RH, particle growth is observed.

**Figure 9:**
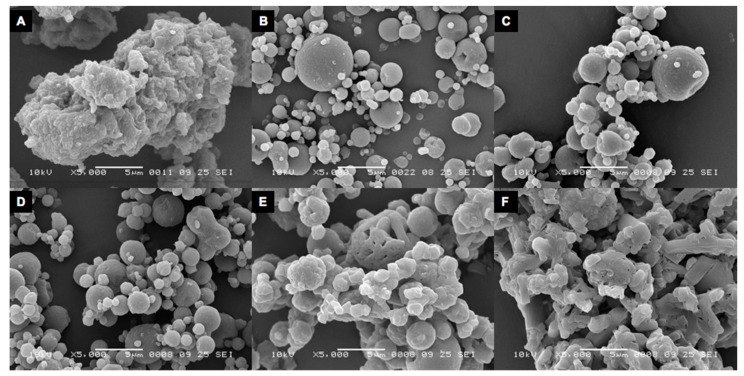
Scanning electron microscopy images of spray-dried isoniazid. (**A**) shows pure spray-dried isoniazid. (**B**) spray-dried isoniazid with 5% l-leucine directly after spray drying. (**C**–**F**) show samples stored for two days at 0%, 58%, 75%, and 90% RH, respectively. Magnification: 5000×.

SEM imaging showed that the isoniazid particles spray dried without excipients consisted of small particles fused together to form the large particles found during the laser diffraction analysis. The addition of 5% l-leucine seemed to prevent such agglomeration, as can be seen in Figure 9B. Storage at 0% RH and 58% RH conserved morphology. However, after storage for two days at 75% RH, the morphology changed to a more fused appearance. This change is even more pronounced for the samples stored at 90% RH.

**Figure 10:**
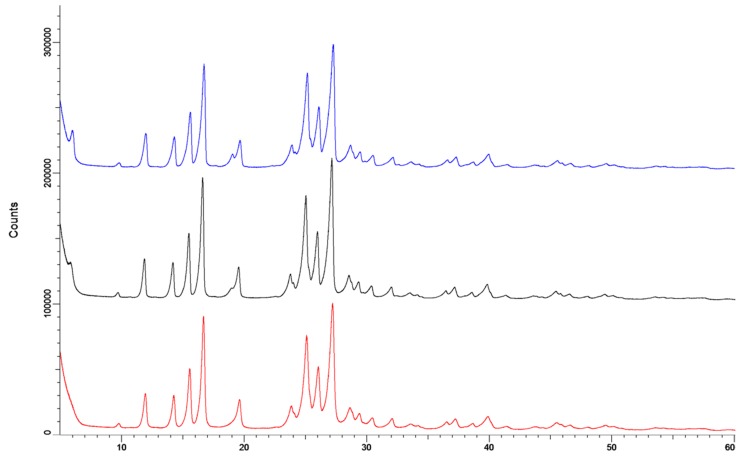
The X-ray spectra of the isoniazid +5% l-leucine samples. The red line is directly after spray drying, the black line is after storage at 0% RH for 48 h and is offset by 100,000 counts. The blue line is after storage at 90% RH and is offset by 200,000 counts.

The X-ray diffraction data (Figure 10) showed no effect of storage. The sample measured directly after spray drying (red line) and after storage at 0% RH (black) showed exactly the same pattern. The diffraction pattern of the sample stored at 90% RH (blue) had some minor differences at 6°2θ and at 19°2θ. However, these differences are too small to indicate a difference in crystal structure. Furthermore, the absence of an amorphous halo indicates that the samples are completely crystalline.

**Figure 11:**
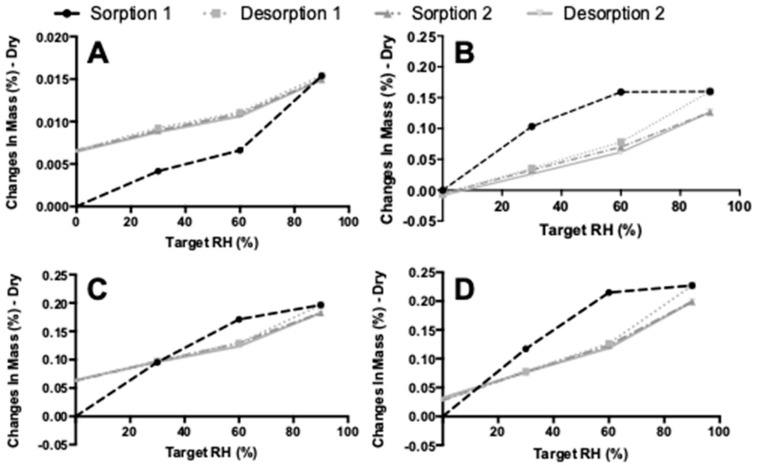
The dynamic vapor sorption (DVS) results. (**A**) shows the starting material results, (**B**) spray-dried isoniazid pure, (**C**) spray-dried isoniazid with 2% l-Leu, and (**D**) shows the spray-dried isoniazid with 4% l-Leu results.

Figure 11 shows the results of the DVS measurements. The first sorption cycle is different for samples that were spray dried. The starting material has a maximum weight gain between 60% and 90% RH. For samples spray dried with or without l-leucine, the weight gain occurs at lower RH values and, in fact, seems to increase with an increasing l-leucine content. The moisture sorption is also higher for spray-dried samples, which may be explained by the increased surface area. It is important to note that the starting material increased only 0.014% in mass, while the increase is around 0.2% for the other samples. Thus, hardly any water is adsorbed in any of the samples at all. Lastly, while the first sorption cycle is different for all four samples, the first desorption cycle and the following second sorption–desorption cycle had the same trend for all samples.

## 4. Discussion

To the best of our knowledge, this is the first time sublimation and Tg values are reported for isoniazid. Isoniazid is shown to lose mass starting at 80 °C. Water evaporation during heating may contribute slightly to the mass loss of isoniazid, but only marginally so considering the low moisture uptake of maximally 0.015%, as measured by DVS for the starting material. With a melting temperature found at 172.23 ± 0.20 °C, this mass loss is, therefore, likely the result of sublimation. Furthermore, isoniazid starting material is shown to be crystalline, with the absence of any indication of amorphous regions, and has a Tg of −3.99 ± 0.18 °C.

Besides performing DVS, an effort was made to determine the water content in the starting materials and subsequent formulations by the use of the Karl Fischer method. However, this proved to be problematic. A single measurement took more than half an hour, while normally this would take a matter of minutes. Furthermore, no reliable duplicates could be generated. This problem may be caused by a reaction of the amide group of isoniazid with the methanol in the reagent. With a methanol free reagent, no reliable duplicates could be generated either. However, based on the DVS results, it seems reasonable to assume that all isoniazid products contain very low amounts of moisture (Figure 11). In the future, it should be investigated whether gas chromatography can be used to ascertain the water content of the isoniazid powders, in an effort to support the DVS findings.

Milled isoniazid without any excipient results in a formulation that blocked the inhaler during most dispersion measurements, which makes this material unsuitable for powder inhalation. The blocking of the inhaler might be the result of the odd particle morphology of the milled material. It was expected to see particles with a shard-like morphology, as jet milling causes collisions between particles, shattering them across crystal planes with the lowest binding energy [18]. However, a fused-sphere morphology was obtained (Figure 3). This morphology may be the result of local increases in temperature during milling, as a result of mechanical stress or shear [19]. This causes some of the isoniazid to sublimate. When sublimated isoniazid cools down, it deposits on the surface of agglomerates and fuses particles together. This binding force between these spheres, however, seems weak enough for RODOS dry dispersion to disperse the powder into primary particles. So, while milling isoniazid results in a primary particle size distribution with a high fraction <5 μm, the blocking of the inhaler makes it necessary to take further formulation steps or to adapt the inhaler.

Adding the lubricant magnesium stearate supports the notion that local increases in temperature during milling and subsequent sublimation and deposition fuse the particles together. Adding a lubricant should lower the local increases in temperature, lower the inhaler retention, and increase the fine particle fraction. Magnesium stearate shows, as expected, a concentration dependent effect on the fine particle fraction and leads to a particle size distribution suitable for inhalation. Inhaler retention dropped following all concentrations of added magnesium stearate except for the B2 batch with 0.5% magnesium stearate, which still blocked the inhalers. Magnesium stearate was added to gain additional insights. The quantity required to reach the desired effect combined with the fact that antibiotics have to be administered in high doses, and that magnesium stearate is poorly absorbed by the body, makes it an undesirable excipient for the actual formulation. It is currently not known whether magnesium stearate could accumulate and disturb the delicate balance existing in the lungs.

The lubricant l-leucine, which might be better tolerated than magnesium stearate, did not result in the same increases in fine particle fraction and lowering of the inhaler retention as magnesium stearate. l-leucine is an essential amino acid that is present in the lungs [20]. This, combined with its higher solubility, makes it a more appropriate excipient for pulmonary administration compared with magnesium stearate. To our knowledge, no maximum concentration of l-leucine is defined by the FDA, EMA, or PDMA. However, in a phase 1 study, 20 subjects were administered a capreomycin formulation containing l-leucine. The l-leucine dose during this study was between 5 and 60 mg. Pulmonary function was unchanged post administration, with mild to moderate side effects, such as cough, being experienced by several subjects [21]. These side effects might have been caused by capreomycin or l-leucine, or both. However, these data indicate that l-leucine is relatively well tolerated. The fraction <5μm was the same for the B1 batch when compared with pure isoniazid, while for B2, it was reduced upon increasing the leucine concentration. Fine particle fractions for both batches were different from the magnesium stearate results. Furthermore, the B2 batch results in a substantially worse performance when compared with B1. As both batches of starting material have the same physicochemical properties, except for the particle size (Figure 1), the particle size may be the reason for the observed differences. Unfortunately, the particles of the unprocessed starting materials were too big to be measured with laser diffraction analysis. When large particles (isoniazid) are mixed with fine powder (l-leucine), an interactive mixture can be formed. In an interactive mixture, fine powder attaches to the larger particles to form some sort of coating [22]. As there is a visual difference between the batches, it is likely that the interactive mixture of these compounds is also different. How this exactly translates to the results found in this study is not known and is an interesting issue to consider for future research. So, while l-leucine is known for lubrication [20,23], and has been successfully used in other milled formulations [24], it does not result in a suitable formulation here.

Adapting the inhaler to the physicochemical properties of pure milled isoniazid did not resolve the blocking problem encountered. As we have described before, for high dose APIs, it is often more beneficial to adapt the inhaler to the API than it is to add excipients to the formulation [25]. The powder inlet channel was increased in width to prevent it from getting blocked. However, this moved the problem to the classifier inlet. The classifier inlet was increased to an extent that did not interfere with the function of the classifier. However, this increase was not sufficient to prevent it from getting blocked. Some other formulation steps are thus still required to get a usable isoniazid DPI product.

Spray-dried pure isoniazid results in particle size distributions that are far too large for pulmonary administration, which is likely a result of unavoidable crystallization in the collection vial. Unexpectedly, some of the particles were larger than the droplets generated in the nozzle, which were around 27 μm. Furthermore, it was the inlet temperature that had the largest influence on the particle size. DSC data show that these powders are crystalline immediately after spray drying. An explanation for these results is that isoniazid crystallizes in the collection vial, which causes fusion of primary particles. This hypothesis is corroborated by the DSC data, which show that isoniazid was completely crystalline immediately after spray drying, something that unfortunately cannot be avoided with a Tg as low as −3.99 ± 0.18 °C. Also, as can be seen in Table 3, before the collection vial and the cyclone, the particle size distribution is substantially smaller and more in line with what is expected. This excludes the possibility that these large particles form while suspended in the drying air. Furthermore, SEM imaging of pure isoniazid showed particles that seem to consist of smaller particles fused together. This might be prevented by increasing the residence time in the spray drier to such an extent that isoniazid is crystallized before it is collected. It is important to note that particle engineering with pure isoniazid is severely limited as the outlet temperature cannot exceed 80 °C (Table 2). After this point, isoniazid will start to sublimate and a similar problem as encountered during milling pure isoniazid may be expected. Particle engineering with excipients is, however, still an option.

The addition of 5% l-leucine to isoniazid results in a spray dried product with a fraction <5 μm suitable for inhalation, and no fusion between particles occurred. The fraction <5 μm is at least four times higher than that found for spray dried isoniazid without excipient. Dispersion measurements showed that the fine particle fraction is quite high with 76.67% ± 0.13%, and that inhaler retention is relatively low with only 9.45% ± 4.57%. The fine particle fraction is 15% higher than that in the best milled isoniazid/l-leucine formulation with a similar retention. It was observed that 50 mg of this spray-dried powder could be filled in the dose compartment of the inhaler, compared with only 25 mg for the milled material.

l-leucine is known to enrich at the surfaces of droplets during spray drying and form a coating on the dry particles [26]. This coating might prevent the isoniazid cores from interacting with each other during the crystallization process, and thus prevent particle fusion. This explanation is corroborated by the fact that no fusion of particles is found in the SEM images. Another possible explanation could be that the addition of l-leucine changes the crystallization behavior of isoniazid. However, this is unlikely as X-ray diffraction shows that no different polymorphs or co-crystals are formed (Figure 10). In prior studies, compounds were spray dried to obtain an l-leucine coating, but never to prevent particle fusion. These studies have either added l-leucine to increase the dispersion efficiency from the inhaler [27,28,29,30,31], or to protect spray-dried powders from moisture [32,33]. This is the first time a l-leucine coating, or a coating in general, has been used to prevent particle fusion after spray drying due to crystallization.

The formulation of isoniazid with 5% l-leucine is not stable when stored under humid conditions for 48 h, and likely suffers from particle fusion as a result of the dissolution–crystallization (Oswald) phenomena. The fraction <5 μm decreased dramatically upon storage at 75% RH and even more so at 90% RH. Figure 6 shows that at higher humidity, more fusion of particles occurred. X-ray diffraction analysis showed that no different crystalline structures were formed, which suggests that all samples are of the same polymorph. On the other hand, DVS analyses showed that something does happen when the powders are exposed to high relative humidity. All spray-dried samples show a higher water uptake in the first sorption cycle compared with the second. Weight increase is most likely from adsorptive water, which changes depending on the total surface area present. When particles fuse together, the available surface area lowers and less water is adsorbed. Furthermore, small particles have a higher packing density than larger ones. In powders with a higher packing density, more capillaries between particles are formed, enhancing capillary condensation and moisture sorption. This effect decreases when particles increase in size [34]. However, water is likely taken up permanently as the starting material sample and the l-leucine samples do not return to the baseline after the first cycle. The exact mechanism by which particles fuse is unknown. It might be solid bridge formation as a result of sintering, dissolution–crystallization, or Ostwald caking [35,36,37,38]. However, sintering is unlikely as the melting point of isoniazid is high (Figure 2). Dissolution–crystallization is the most likely mechanism, as particle fusion is induced by the presence of moisture.

The sensitivity of isoniazid with 5% l-leucine formulation to moisture was unexpected and makes processing and distribution at an industrial scale difficult. It is especially unexpected as l-leucine has previously been used for moisture protection in spray dried formulations [32,33]. While Figure 8 shows that storage for two days at 58% RH or less is possible, the 75% RH and 90% RH results show that the rate of fusion is RH dependent. It is likely that there is a threshold humidity under which no fusion will occur. In follow-up research, this threshold humidity needs to be established, as this is the humidity under which the product should be manufactured and packaged.

While the milled isoniazid with l-leucine can likely be optimized by the use of a second mill step to break up the agglomerates found, or by milling pure isoniazid first and only later adding the lubricant, we feel it is safe to say that the isoniazid spray dried with 5% l-leucine formulation shows the most promise and is the best candidate for further development. Furthermore, optimizing this formulation might raise the threshold humidity, easing the likely strict humidity conditions under which manufacturing and packaging should occur.

Further research is needed, not only to explain the relationship of water and the spray-dried isoniazid- l-leucine powders, but also to see if the dose can be increased, if l-leucine content can be decreased by improving the coating efficiency during the spray drying process, and if it remains stable during prolonged storage. Furthermore, while isoniazid is a highly soluble compound, with a solubility of 140 mg/mL, indicating that any drug administered will immediately dissolve in the mucus or alveolar lining fluids, further research should ascertain this empirically.

## 5. Conclusions

This study set out to physiochemically characterize isoniazid and to use this information to formulate isoniazid for pulmonary administration with little or no excipients. Particle engineering options are limited for isoniazid as a result of its low Tg and thanks to sublimation. The dispersion of pure jet milled isoniazid from the inhaler is poor and is only marginally increased by the use of l-leucine. Magnesium stearate improved dispersion, but only in relatively high concentrations, which might be undesirable for use in patients. Spray drying of pure isoniazid results in a particle size distribution unsuitable for inhalation because of the fusion of particles after collection. Particle fusion was prevented by the addition of 5% l-leucine, and thus resulted in a suitable formulation. However, these l-leucine containing formulations are sensitive to moisture levels over 58% RH. The physical stability of the formulations during prolonged storage and their dispersibility from a dry powder inhaler should thus be studied further.

## Figures and Tables

**Table 1:** Spray drying conditions used with the B-290 mini spray dryer. *N* = 2.

Condition Number	Concentration Isoniazid (mg/mL)	Inlet Temperature (°C)	Feed Rate (ml/min)	Atomizing Air Flow (mm)	Aspirator Air Flow (%)
1	50	60	2.5	50	100
2	50	40	1	50	100
3	50	160	2.5	50	100
4	50	160	12.5	50	100
5	25	60	2.5	50	100
6	75	60	2.5	50	100

**Table 2:** The thermogravimetric analysis (TGA) data showing the temperature and the mass loss at that temperature. Mass loss was measured over four-hour periods.

Temperature	80 °C	100 °C	120 °C	140 °C
Mass loss (%)	0.06 ± 0.02	0.62 ± 0.06	4.29 ± 0.51	24.12 ± 3.24

**Table 3:** Laser diffraction data of spray-dried pure isoniazid during spray drying and after collection (*N* = 2).

Particle Size Distribution	During Spray Drying	After Collection
**X10 (µm)**	1.27 ± 0.06	3.11 ± 0.74
**X50 (µm)**	2.58 ± 0.15	25.13 ± 5.49
**X90 (µm)**	5.34 ± 0.88	111.39 ± 5.53

## Supplementary Materials

The following are available online at https://www.mdpi.com/1999-4923/11/5/233/s1, Figure S1: Representative DSC data showing that all samples were crystalline.
