# Supplementary material for: Characterization and Formulation of Isoniazid for High-Dose Dry Powder Inhalation
Source: Pharmaceutics. 2019 May 13;11(5):233. doi: 10.3390/pharmaceutics11050233 (PMC6572553; doi:10.3390/pharmaceutics11050233)
Supplement: Supplementary file 1 [file pharmaceutics-11-00233-s001.pdf]

pharmaceutics

Supplementary Materials: Characterization and Formulation of Isoniazid for High-Dose Dry Powder Inhalation

Imco Sibum, Paul Hagedoorn, Henderik W. Frijlink and Floris Grasmeijer

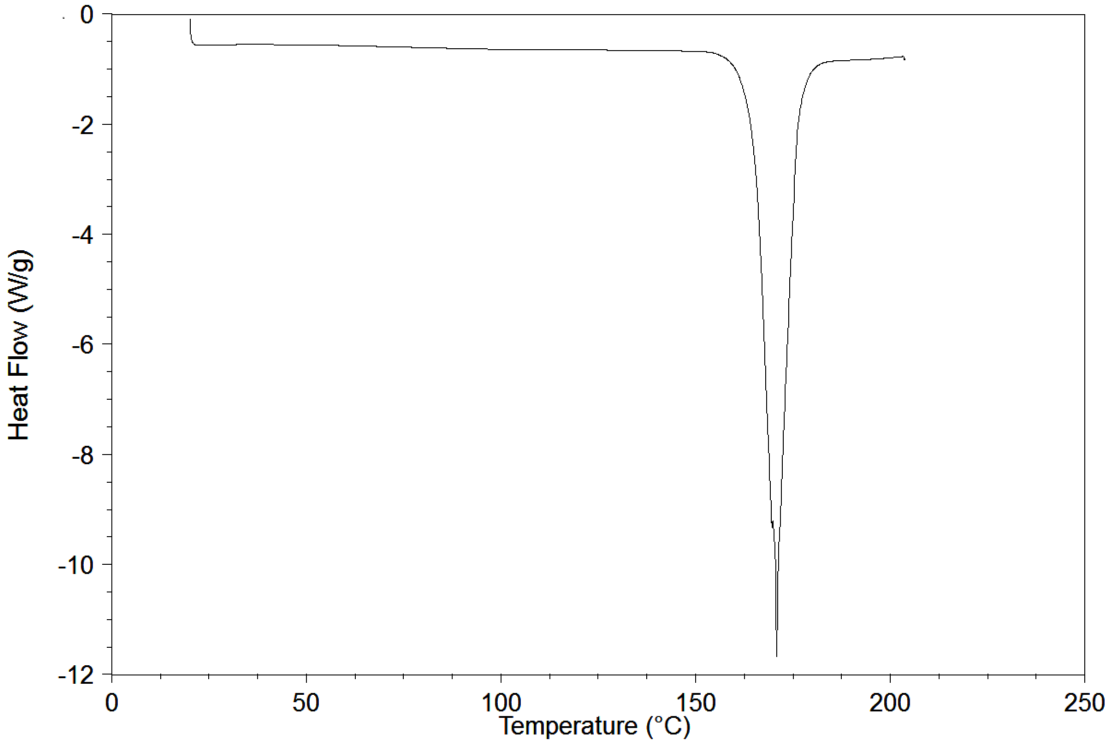

Figure S1. Representative DSC data showing that all samples were crystalline.
